# Comparison of mesenchymal stromal cells from peritoneal dialysis effluent with those from umbilical cords: characteristics and therapeutic effects on chronic peritoneal dialysis in uremic rats

**DOI:** 10.1186/s13287-021-02473-9

**Published:** 2021-07-13

**Authors:** Yangchun Du, Ming Zong, Qiunong Guan, Zhongli Huang, Lan Zhou, Jing Cai, Gerald da Roza, Hao Wang, Hualin Qi, Yiping Lu, Caigan Du

**Affiliations:** 1 Department of Urology, Institute of Urology, West China Hospital of Sichuan University, No. 17, Section 3, Ren Min Nan Road, Chengdu, 610041 China; 2 Organ Transplantation Center, Sichuan Provincial People’s Hospital, University of Electronic Science and Technology of China, Chengdu, China; 3 Department of Urologic Sciences, University of British Columbia, 2660 Oak Street, Vancouver, BC V6H 3Z6 Canada; 4 Shanghai East Hospital, Tongji University School of Medicine, Shanghai, China; 5 Division of Nephrology, Department of Medicine, University of British Columbia, Vancouver, BC Canada; 6 Department of General Surgery, Tianjin Medical University General Hospital, Tianjin, China; 7 Department of Nephrology, Shanghai Pudong New Area People’s Hospital, No. 490 Chuanhuan South Road, Pudong New Area, Shanghai, 201299 China

**Keywords:** Peritoneal dialysis, Peritoneal injury, Peritoneal dialysis effluent-derived mesenchymal stromal cell, Cell therapy

## Abstract

**Background:**

A long-term of peritoneal dialysis (PD) using a hypertonic PD solution (PDS) leads to patient’s peritoneal membrane (PM) injury, resulting in ultrafiltration failure (UFF) and PD drop-out. Our previous study shows that PD effluent-derived mesenchymal stromal cells (pMSCs) prevent the PM injury in normal rats after repeated exposure of the peritoneal cavity to a PDS. This study was designed to compare the cytoprotection between pMSCs and umbilical cord-derived MSCs (UC-MSCs) in the treatment of both PM and kidney injury in uremic rats with chronic PD.

**Methods:**

5/6 nephrectomized (5/6Nx) Sprague Dawley rats were intraperitoneally (IP) injected Dianeal (4.25% dextrose, 10 mL/rat/day) and were treated with pMSCs or umbilical cord (UC)-MSCs (approximately 2 × 10^6^/rat/week, IP). Ultrafiltration was determined by IP injection of 30 mL of Dianeal (4.25% dextrose) with 1.5-h dewell time, and kidney failure by serum creatinine (SCr) and blood urea nitrogen (BUN). The structure of the PM and kidneys was assessed using histology. Gene expression was examined using quantitative reverse transcription PCR, and protein levels using flow cytometric and Western blot analyses.

**Results:**

We showed a slight difference in the morphology between pMSCs and UC-MSCs in plastic dishes, and significantly higher expression levels of stemness-related genes (*NANOG*, *OCT4*, *SOX2*, *CCNA2*, *RAD21*, and *EXO1*) and MSCs surface markers (CD29, CD44, CD90 and CD105) in UC-MSCs than those in pMSCs, but no difference in the differentiation to chondrocytes, osteocytes or adipocytes. pMSC treatment was more effective than UC-MSCs in the protection of the MP and remnant kidneys in 5/6Nx rats from PDS-induced injury, which was associated with higher resistance of pMSCs than UC-MSCs to uremic toxins in culture, and more reduction of peritoneal mesothelial cell death by the secretome from pMSCs than from UC-MSCs in response to PDS exposure. The secretome from both pMSCs and UC-MSCs similarly inactivated NOS2 in activated THP1 cells.

**Conclusions:**

As compared to UC-MSCs, pMSCs may more potently prevent PDS-induced PM and remnant kidney injury in this uremic rat model of chronic PD, suggesting that autotransplantation of ex vivo-expanded pMSCs may become a promising therapy for UFF and deterioration of remnant kidney function in PD patients.

**Supplementary Information:**

The online version contains supplementary material available at 10.1186/s13287-021-02473-9.

## Background

Peritoneal dialysis (PD) is an effective renal replacement therapy for end-stage renal disease (ESRD), and this therapy has increased in popularity recently [1]. During PD, a PD solution (PDS), such as glucose-based hypertonic Dianeal (1.5 to 4.25% of glucose), is instilled into patient’s peritoneal cavity. After 4 to 6 h (dwell time) of absorbing the waste substances (e.g, uremic toxins) from the blood vessels within the peritoneum, the resultant PD effluent is drained out from the cavity and discarded. As compared to hemodialysis, the benefits of using PD include technical simplicity or minimal requirement for medical staff support, more cost-effectiveness, reduced dietary restriction, better protection of residual renal function (RRF), improved quality of life, and lower doses of erythropoietin required [1–3].

However, many factors cause chronic peritoneal inflammation in PD patients [4, 5]. Firstly, the accumulating uremic toxins such as dietary advanced glycation endproducts (AGEs), from increased formation and decreased renal clearance in PD patients, trigger oxidative stress, and inflammation via activation of AGE receptor in the peritoneal membrane (PM) [6, 7]. Secondly, PD catheter causes peritonitis or infection [8] that activates resident and infiltrating immune cells (e.g., macrophages) and stimulates the release of proinflammatory cytokines (e.g., IL-1 and IL-6) or growth factors (e.g., TGF-β and FGF) in the peritoneal cavity [9, 10]. Thirdly, the bioincompatibility of PDS (acidic pH, hyperosmolality, glucose degradation products, and lactate) also induces biochemical cascade complex, including cell death, AGE generation from the glucose degradation products, oxidative stress, and resident (myo)fibroblast activation that accelerate the peritoneal inflammation (IL-1, IL-6, and TNF-α), resulting in PM damage or thickening [11, 12]. Taken together, the presence of chronic inflammation, particularly in the PM of PD patients, results in the structural alterations in the peritoneum including tissue fibrosis and neoangiogenesis [13, 14], and consequently ultrafiltration failure (UFF) that occurs in up to 50% of PD patients after 6 years of PD [15, 16]. UFF is one of main reasons for PD drop-out [17, 18]. Therefore, successful control of PD-induced chronic peritoneal inflammation or preservation of the tissue structure of the peritoneum during the PD could prolong the PD therapy the ESRD patients need.

Mesenchymal stromal cells (MSCs), also known as mesenchymal stem cells, are characterized by the spindle or fibroblast-like plastic adherence, a unique profile of cell surface marker expression (positive for CD29, CD44, CD73, CD90, CD105, CD106, CD166, and Stro-1; negative for CD45, CD34, CD14, CD79, and HLA-DR), and multilineage differentiation into mesodermal cell types under specific culture conditions [19, 20]. MSCs have been successfully isolated from the bone marrow (BM), dental pulp, hair follicles, placenta and umbilical cord (UC), adipose tissue, synovium, and testis [21–23]. We have also identified the MSCs in otherwise discarded PD effluent for the first time [24], and unlike the MSCs from other sources, these PD effluent-derived MSCs (pMSCs) are negative for CD105, Stro-1, and SSEA-4, but positive for CD200 [24, 25]. Accumulating data from both experimental models and clinical trials during the past decades have been showing the immunoregulatory effects of MSC-based therapies on the treatment of inflammation and tissue damage [26–28]. These findings may imply the therapeutic potential of MSCs for treatment of chronic peritoneal inflammation or peritoneum damage in PD patients. Indeed, the treatment with UC-derived MSCs (UC-MSCs) significantly reduces PDS-induced tissue injury (submesothelial thickness), inflammation, angiogenesis, and fibrosis of peritoneal membrane (PM), resulting in preventing UFF in a rat model of PD [29]. Recently, we also report the similar effectiveness of pMSCs on PDS-induced PM injury and PM dysfunction in rats [30]. Furthermore, a small phase I clinical trial with 10 PD patients shows that infusion of autologous adipose tissue-derived MSCs (AD-MSCs) via cubital vein decreases the rate of solute transport (creatinine) across the PM without both the serious adverse effects and catheter-related complications in PD patients [31]. As compared with the UC-MSCs or AD-MSCs that may be restricted by the invasive harvest procedure or ethical concerns, pMSCs may offer an unlimited and promising source of MSCs as a patient-specific medical product specifically for clinical use in PD patients. The objective of this study was to compare the effectiveness between UC-MSCs and pMSCs on the protection of PM and remnant kidney of uremic rats from PDS-induced injury and dysfunction.

## Methods

### Animals and cells

Subtotal 5/6 nephrectomized (5/6Nx) Sprague Dawley rats (male, 12–14-week old, bodyweight 250–350 g) were purchased from the Charles River Laboratories International, Inc. (Surgery Code: 56NEPHREX, Wilmington, MA, USA). Immortalized human peritoneal mesothelial cells (HPMCs) were generated in our laboratory and were grown in K1 complete culture medium (K1 medium) as described previously [32]. THP-1 cells, a human monocytic cell line (ATCC TIB-202), were grown in Roswell Park Memorial Institute (RPMI)-1640 medium containing 10% of fetal bovine serum (FBS) (Thermo Fisher Scientific, Ottawa, ON, Canada). Both types of cells were expanded and used for the experiments in a 5% CO_2_ humidified incubator at 37 °C.

### Isolation and growth of pMSCs and UC-MSCs

PD effluents were collected after obtaining informed consent from anonymized patients who received Dianeal or Physioneal PD solution-based PD therapy within 4 weeks (Table 1), following the approved protocol H15-02466 (Office of Research Ethics/Clinical Research Ethics Board, The University of British Columbia, Vancouver, BC, Canada). The pMSCs in the PD effluents were simply pelleted by the centrifugation and were expanded in Dulbecco’s modified Eagle’s medium/Ham’s nutrient mixture F12 (DMEM/F12) containing 10% FBS (Thermo Fisher Scientific) in plastic culture dishes as described previously [24]. The UCs were collected from the BC Children’s Hospital Biobank (Vancouver, BC, Canada), and no any donor information including parents and babies was collected due to the ethical concerns. For this study, the UCs were collected from both healthy girls and boys (excluding premature babies), and there were no complications during pregnancy and at birth. UC-MSCs were isolated from Wharton’s jelly of the UC as described previously [33]. In brief, after removing arteries and veins, Wharton’s jelly was transferred to the plastic culture dishes and was minced into small pieces or explants. The explants were incubated in the 10% FBS-DMEM/F12 medium in a humidified CO_2_ (5%) incubator at 37 °C until MSCs grew and migrated from the explants. The culture medium for the growth of both pMSCs and UC-MSCs was changed every 3 days, and the remaining adherent cells (70–80% confluence) were passaged by a brief incubation with 0.25% GIBCO Trypsin/EDTA solution (Thermo Fisher Scientific). After four passages (P4), both pure pMSCs and UC-MSCs (approximately 10^6^ cells/vial) were frozen with 10% dimethylsulfoxide (DMSO) in liquid nitrogen for following in vitro and in vivo experiments.

**Table 1:** The demographic Information of donors

Donor number	Age range (yr)	Gender code	NIH ethnicity categories	Time on PD (wk)	PD solution
1	30-39	0	Asian	The first PD	Dianeal
2	50-59	1	Asian	The first PD	Dianeal
3	60-69	1	White	The first PD	Dianeal
4	80-89	1	Asian	The first PD	Physioneal
5	30-39	0	Asian	The 3^rd^ wk	Physioneal
6	60-69	0	White	The first PD	Dianeal
7	60-69	1	White	The first PD	Dianeal
8	30-39	0	Asian	The 2^nd^ wk	Dianeal
9	80-89	0	White	The first PD	Dianeal

The PD effluents were collected under protocol H15-02466 approved by the Clinical Research Ethics Board at the University of British Columbia. The gender code for female or male is available from the corresponding authors upon request

### Quantitative reverse transcription-polymerase chain reaction (RT-qPCR)

The mRNA expression of a panel of stemness markers was examined using real-time RT-qPCR and their specific primer pairs (Suppl. Table 1). Total RNA was extracted from pMSCs or UC-MSCs at P4 by using mirVana™ isolation kit (Ambion, Austin, TX, USA). Only the RNA samples with ≥ 8 of RNA Integrity Number were used for RT-qPCR analysis. In brief, 1 μg of high quality total RNA from each sample was reverse transcribed to cDNA by using RT^2^ First Strand Synthesis Kit (QIAGEN, Toronto, ON, Canada). The cDNA of a target gene was then amplified by real-time PCR using KiCqStart Probe qPCR ReadyMix^TM^ (Product No. KCQS06) and corresponding primer pair (Suppl. Table 1) with PCR amplification conditions (3 min at 95 °C, followed by 40 cycles of 15 s at 95 °C and 60 s at 60 °C) according to the manufacturer’s instruction (Sigma-Aldrich Canada, Oakville, ON, Canada).

The raw qPCR values of both housekeeping gene *β-actin* and the marker genes in each sample were initially collected on the threshold cycle (Ct). A difference as a ΔCt value between the Ct values of a marker gene and the housekeeping *β-actin* was calculated, followed by the calculation of the double delta Ct value (ΔΔCt), a difference between the ΔCt of the marker gene in a test sample and the average ΔCt of this gene of all the samples (as an average control). Finally, the expression fold change of the marker gene in a test sample relative to the average control was calculated and presented as a 2^−ΔΔCt^ value (2 to the power of negative ΔΔCt) as described previously [34].

### Determination of cell surface expression of MSC markers

The cell surface expression of a panel of MSC markers (CD14, CD29, CD34, CD44, CD45, CD73, CD79a, CD90, CD105, CD146, CD166, CD271, HLA-DR, SSEA-4, and Stro-1) on both pMSCs and UC-MSCs from the passages of 4 to 7 was measured by using fluorescence-activated cell sorting (FACS) analysis as described previously [24]. The fluorescent-conjugated monoclonal antibodies for this analysis were purchased from *e*Bioscience (San Diego, CA, USA), Biolegend (San Diego, CA, USA), or BD Biosciences (Mississauga, ON, Canada) (Suppl. Table 2). In brief, a single-cell suspension of pMSCs or UC-MSCs from the same passage was stained with the antibodies in the dark for 30 min at 4 °C. After washing with PBS, the mean fluorescence intensity (MFI) of the stain of each cell surface marker was determined using a calibur flow cytometer (BD Biosciences). Data were analyzed with FlowJo software (FlowJo, LLC., Ashland, OR, USA).

### Trilineage differentiation

The differentiation of pMSCs or MC-MSCs from passage 4 to 7 was induced to chondrocytes, osteocytes, or adipocytes by incubation in a high-glucose DMEM medium containing different mixtures of supplements as described previously [19, 24, 35]. The differentiated chondrocytes were confirmed by the presence of acidic Alcian blue-stained cartilage formation, the Ca_2_^+^ matrix mineralization in the osteocytes was stained with Alizarin red S, and the lipid droplets inside the adipocytes were stained positively by the Oil Red O dye.

### Preparation of MSC-conditional medium (CM) for in vitro tests

The preparation of pMSCs-CM or UC-MSC-CM was described previously [30]. In brief, the frozen MSCs after thaw were grown to be 80–90% confluent in plastic culture dishes, followed by a 24-h incubation period with approximately 5 mL of the culture medium per 10^6^ cells. At the end of the incubation, the cellular debris in the medium was pelleted by centrifugation at 12,000×*g* for 10 min at 4 °C, and resultant supernatant was harvested as pMSCs-CM or UC-MSCs-CM.

### Chronic PD in uremic rats, and MSC preparation and transplantation

Chronic PM injury in 5/6Nx rats was induced by intraperitoneal (IP) injection (10 mL/day/rat) of 4.25% dextrose PDS (Dianeal, 484 mOsmol/L, pH 5.2) (Baxter Healthcare, IL, USA) for a period of 6 weeks as described previously [30]. A large quantity of both pMSCs and UC-MSCs for in vivo treatment was prepared as described in our previous study [30]. In brief, frozen cells were rapidly thawed and washed once with the culture medium. The washed cells were grown in the medium in the plastic petri dishes in a 5% CO_2_ humidified incubator at 37 °C until the cell culture reached 70–80% confluent (during log phase of growth) as described above. Adherent MSCs were harvested by trypsinization and were suspended in phosphate buffered saline (PBS) (2 × 10^6^ MSCs per mL of PBS) for rat treatment.

Four experimental groups were included in this study. Group 1 (PBS control), rats (*n* = 5) received daily PBS injection (10 mL/day/rat) only. Group 2 (PDS), rats (*n* = 7) received daily PDS and treatment with PBS vehicle (IP, 1 mL/rat/week, started at day 1). Group 3 (PDS + pMSCs), rats (*n* = 7) received daily PDS and pMSC treatment (IP, 2 × 10^6^ cells/rat/week, started at day 1). Group 4 (PDS + UC-MSCs), rats (*n* = 7) received daily PDS and UC-MSC treatment (IP, 2 × 10^6^ cells/rat/week, started at day 1). The PDS and MSCs or vehicle were administrated at different times; the PDS in the early morning (9 am–10 am), and MSC injection in the late afternoon (5 pm–6 pm). The primary outcome measure was the effects of MSC treatment (pMSCs and UC-MSCs) on PM injury. The secondary measure was their effects on remnant kidney injury.

### Urine specimen collection

The urine specimens were collected prior to the endpoint of MSC treatment by using a metabolic cage. In brief, a single rat was housed in the metabolic cage and was fed ad libitum on the same food and drink water as in the “home” cages. The urine was collected during 8 h of the dark phase in a 12-h light/dark cycle at 25 °C, followed by centrifugation at 5000×*g* to pellet cellular debris. The supernatant (urine) was stored in aliquots at − 80 °C until use.

### Measurement of glucose, creatinine, and blood urea nitrogen

The levels of glucose (GLU), total protein, creatinine (Cr), and blood urea nitrogen (BUN) in the fluid samples (urine, dialysate, plasma, and serum) were determined using the Dimension Vista® 1500 System (Siemens Healthineers Canada, Oakville, ON, Canada) in the Clinical Chemistry Laboratory at the Vancouver Coastal Health Regional Laboratory Medicine (Vancouver, BC, Canada).

### Function parameters of the PM and kidney

The PM function was determined using four parameters of the peritoneal permeability or solute transport: ultrafiltration (UF), dialysate-to-plasma ratio (D/P) of GLU, and clearance of both Cr (C_Cr_) and BUN (C_BUN_) as a primary outcome of different MSCs therapies in this study. In brief, at the end of 6-week MSC treatment, 30 mL of Dianeal (4.25% dextrose) was slowly IP injected to each rat, and it was allowed to dwell in the peritoneal cavity for 90 min. The dialysate was recovered from the cavity using a syringe as much as possible, and the plasma isolation from the blood was done by using EDTA-containing tubes and centrifugation (1500×*g*, 10 min at 4 °C). UF was the volume (mL) of dialysate recovered from the cavity. Solute clearance (C_Cr_ and C_BUN_) was calculated by multiplying the D/P ratio of a solute with the dialysate volume (D/P × V). The levels of serum Cr (SCr) and BUN, and the protein-to-Cr ratio (PCr) in urine samples were used to measure the kidney function in 5/6Nx rats after MSC treatment.

### Histopathological assessment of PM and remnant kidneys

At the end of MSC treatment, the structural changes in both PM and remnant kidneys were examined by YD, QG, and CD in a blinded fashion using histopathological assessment as described below. In brief, two pieces of tissues on the opposite site of anterior parietal peritoneum from the injection site were collected from each rat, and the remnant kidney was split in the median longitudinally. Both peritoneal and kidney tissues were fixed in formalin (10%) and embedded in paraffin. The tissue sections (4 μm) were stained with hematoxylin and eosin (H&E) and were scanned using Leica SCN400 Slide scanner (Leica Microsystens Inc., Concord, ON, Canada). The images of both the PM and the renal cortex were examined by using the Digital Image Hub – A slidepath Software Solution (Leica Microsystems Inc.). In the PM sections, the tissue damage was determined by the changes of both submesothelial thickness, a space from the inner surface of the muscle to the mesothelium, and the number of the blood vessels or capillaries within the submesothelial layer as previously described [30, 36]. Fifteen to twenty high-powered fields of each PM section (two per a rat) were randomly selected for histological examination, and an average number of all the measures represented the pathological score of an individual animal. In the kidney sections, the number of dilated tubules, including intraductal cast formation, atrophy (cell loss), and tubular cell flattening, as a mark of kidney damage was counted in each view of the renal cortical region under × 100 magnification. An average number of 20 to 30 randomly selected, non-overlapping high-powered fields represented the number of injured tubules in each kidney.

### Lactate dehydrogenase (LDH) release assay

The sensitivity of pMSCs compared to UC-MSCs to the toxicity of a panel of uremic toxins was determined by the levels of LDH release. In brief, 0.2 × 10^6^ pMSCs or UC-MSCs were seeded per well in 24-well plates overnight, followed by incubation with culture medium containing different mixtures of uremic toxins (Suppl. Table 3) in a 5% CO_2_ incubator at 37 °C for 24 h. The pMSCs or UC-MSCs incubated with 0.2% Triton X-100 were used as a positive control (100% of cell death). The cellular debris in the resultant supernatant was pelleted by centrifugation at 10,000×*g* for 5 min, and the levels of LDH in the supernatants were determined by using the Cytotoxicity Detection Kit (LDH) (Roche) (Sigma-Aldrich Canada) following the manufacturer’s protocol. The cell death of pMSCs or UC-MSCs in response to the uremic toxin mixture was calculated as follows: LDH release (%) = sample OD_450_/positive control OD_450_ × 100%.

### Measurement of cytoprotection of pMSCs-CM or UC-MSCs-CM in vitro

The cytoprotective activity of pMSCs-CM as compared to that of UC-MSCs-CM was determined in cultured HPMCs after a brief direct exposure to a hypertonic PDS, PDS-induced cell death. A monolayer of HPMCs after overnight incubation with K1 medium was treated with the culture medium only (Medium group) or with Dianeal (4.25% dextrose) for 20 min. After 20 min treatment, these PDS-treated cells were further treated with the culture medium only (100% culture medium, PDS group) or the medium containing either 50% (v/v) pMSCs-CM (PDS + pMSCs) or 50% (v/v) UC-MSCs-CM (PDS + UC-MSCs) in a 5% CO_2_ incubator at 37 °C for 24 h. The cell apoptosis or viability was quantitatively determined by using FACS analysis with double staining of Annexin-V conjugated with phycoerythrin (Annexin-V-PE) and 7-amino-actinomycin D (7-AAD) as described in our previous study [37].

### Inactivation of monocytes/macrophages by pMSCs-CM or UC-MSCs-CM in vitro

Human THP-1 monocyte cultures (approximately 10^5^ cells/mL) were activated or induced to macrophage differentiation by addition of phorbol 12-myristate 13-acetate (PMA) first, followed by additional stimulation with lipopolysaccharides (LPS) as described previously [30]. The activated THP-1 cells were subsequently treated with the culture medium alone (100% culture medium, PMA/LPS group) or the medium containing either 50% (v/v) pMSCs-CM (PMA/LPS + pMSCs) or 50% (v/v) UC-MSCs-CM (PMA/LPS + UC-MSCs) in a 5% CO_2_ incubator at 37 °C for 24 h. The unstimulated THP1 cells after 24 h incubation with the culture medium were used as a baseline control (Medium group).

### Nitric oxide (NO) measurement

After 24 h of treatment with pMSCs-CM, UC-MSCs-CM, or medium control, the levels of nitrite as a product of NO by oxidation in the supernatant were measured using the Griess method [30]. In brief, 50 μL of culture supernatant were first incubated with 50 μL of 1% sulfanilamide in 5% phosphoric acid (96-well plates in triplicates) for 10 min, followed by addition of 50 μL/well of 0.1% naphthylethyline diamine dihydrochloride. The color development was quantitatively measured at 550 nm, and the level of NO/nitrite in each sample was calculated using a standard curve with known sodium nitrite concentrations.

### Western blot analysis

After 24 h of treatment with pMSCs-CM, UC-MSCs-CM, or medium control, total cellular protein extracts from THP-1 cells were harvested, and the protein levels of nitric oxide synthase 2 (NOS 2) were determined by Western blot as described previously [30, 38]. In brief, the cellular protein extracts (approximately 100 μg protein/sample) were fractionated by 7% SDS-polyacrylamide gel electrophoresis (SDS-PAGE) and were transferred onto a nitrocellulose membrane. NOS 2 protein bands were specifically detected by primary rabbit polyclonal anti-NOS 2 antibody (N-20) (Santa Cruz Biotech, Santa Cruz, CA, USA) and secondary goat anti-rabbit IgG antibody (Vector Lab., Burlingame, CA, USA). The blots were re-probed using anti-β-actin (Sigma-Aldrich Canada) for confirmation of the amount of loaded protein in each sample.

### Statistical analysis

Data were presented as the mean ± standard deviation (SD). The differences between groups were compared by using analysis of variance (ANOVA) or *t*-tests (two-tailed distribution) of Prism GraphPad software version 4 (GraphPad Software, Inc., La Jolla, CA, USA) as appropriate. A *p* value of ≤ 0.05 was considered significant.

## Results

### Differences of MSC characteristics between pMSCs and UC-MSCs

**Fig. 1:**
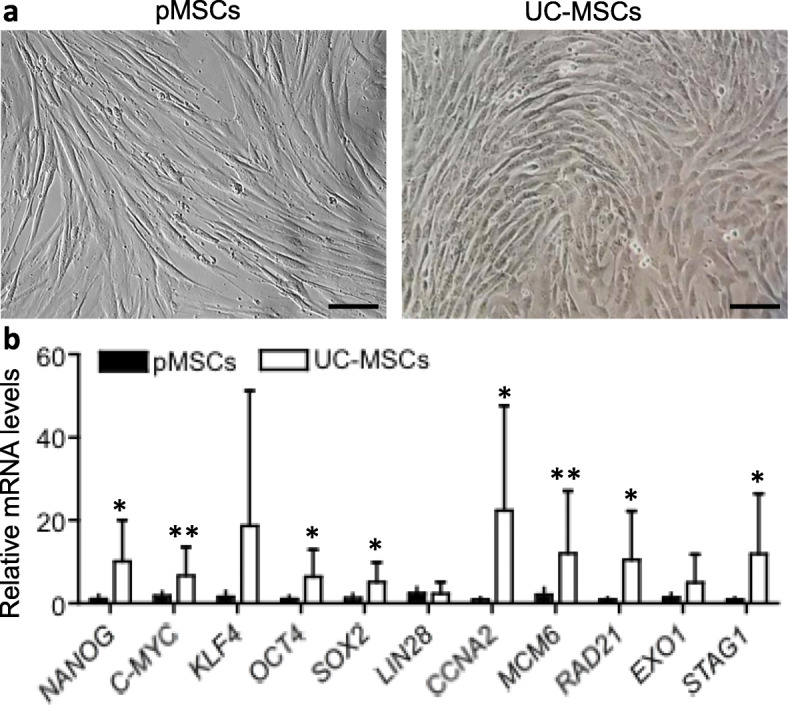
Morphology and stemness marker gene expression between pMSCs and UC-MSCs at P4. **a** A typical microscopic image of a confluent monolayer of pMSCs or UC-MSCs growing in plastic tissue culture plates. pMSCs displayed a spindle shape, and UC-MSCs cobble-stone like morphology. Bar: 100 μm. **b** The expression (relative mRNA) of a panel of stemness marker genes, which was presented as fold changes (2^−ΔΔCt^) and determined using RT-qPCR as compared to the internal *β-Actin* control. Data are presented as the mean ± standard deviation (SD). The different expression of a gene in between pMSCs (*n* = 9 donors) and UC-MSCs (*n* = 7 donors) was analyzed using two-tailed *t*-test. *Statistically significant (*NANOG*, *p* = 0.0169; *OCT4*, *p* = 0.0284; *SOX2*, *p* = 0.034; *CCNA2*, *p* = 0.0223; *RAD21*, *p* = 0.0272; and *EXO1*, *p* = 0.039), **marginally significant (*C-MYC*, *p* = 0.0595; *MCM6*, *p* = 0.0648). This experiment was repeated three times in triplicate using independently prepared cDNAs, and the results were exhibited in almost identical patterns

The differences in the microscopic morphology, expression of stemness genes and MSC surface markers, and multipotential differentiation between pMSCs and UC-MSCs were compared under the same conditions. As shown in Fig. 1a, both pMSCs and UC-MSCs equally adhered to and expanded on the surface of plastic tissue culture plates in cultures. The morphology of pMSCs was characterized by a spindle shape, whereas UC-MSCs displayed more the characteristic cobble-stone or “fibroblast” morphology. The stemness of pMSCs and UC-MSCs was determined by the relative mRNA expression of a panel of “stemness markers”—*NANOG*, *C-MYC*, *KLF4*, *OCT4*, *SOX2*, *LIN28*, *CCNA2*, *MCM6*, *RAD21*, *STAGE1*, and *EXO1* to *β-Actin* using RT-qPCR. The Ct (cycle threshold) values of all the target genes except NANOG and LIN28 were under 30 cycles (strong positive). The Ct of *NANOG* was 32 to 36 cycles in pMSCs and 33 to 38 cycles in UC-MSCs (moderate levels), and the expression of *LIN28*was not detectable (Ct > 40 cycles) in 3 of 9 pMSCs samples (the remaining 31–33 cycles) and 2 of 7 UC-MSCs samples (the remaining 34–39 cycles) (weak levels). The relative expression of a target gene was presented by the 2^−ΔΔCt^ to *β-Actin* in each sample (Fig. 1b). The statistical analysis suggested that the expression of *NANOG* (*p* = 0.0169), *OCT4* (*p* = 0.0284), *SOX2* (*p* = 0.034), *CCNA2* (*p* = 0.0223), *RAD21* (*p* = 0.0272), and *EXO1* (*p* = 0.039) was significantly higher in UC-MSCs (*n* = 7) than those in pMSCs (*n* = 9), and the levels of *C-MYC* (*p* = 0.0595) and *MCM6* (*p* = 0.0648) were quite higher in UC-MSCs than pMSCs. The remaining genes (*KLF4*, *LIN28*, and *STAG1*) were not different.

**Fig. 2:**
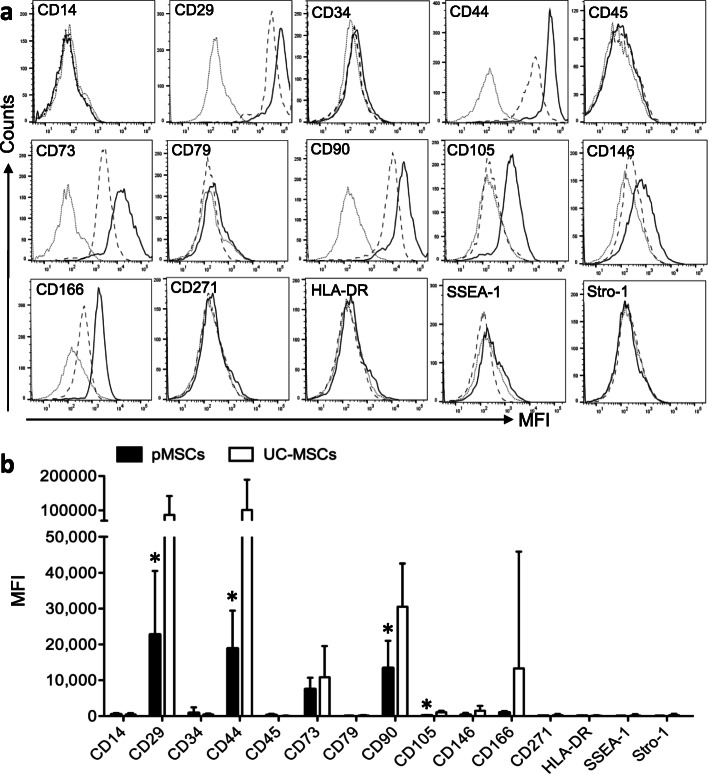
Expression of a panel of MSC typical cell surface markers between pMSCs and UC-MSCs at P4. **a** A typical FACS histogram of each marker expression. A faint line: background staining; a dot line: pMSCs; and a slid line: UC-MSCs. MFI: mean fluorescence intensity. **b** Data are representative of two independent experiments and are presented as the mean ± SD. The different expression of a cell surface marker in between pMSCs (*n* = 9 donors) and UC-MSCs (*n* = 7 donors) was analyzed using two-tailed *t*-test. *Statistically significant (CD29, *p* = 0.0167; CD44, *p* = 0.0422; CD90, *p* = 0.0096; CD105, *p* = 0.0058)

The expression profile of a panel of MSC cell surface markers between pMSCs (*n* = 6) and UC-MSCs (*n* = 9) was also compared. Figure 2a presents a typical histogram of each marker in FACS analysis in both groups. As shown in Fig. 2b, a high level or strong positive (MFI ≥ 2000) was seen in the expression of CD29, CD44, CD73, or CD90 in both groups and of CD166 in 3 of 9 UC-MSC samples, and a low to moderate level (MFI 400–2000) in CD105, CD146, and CD166 in UC-MSCs and CD146 and CD166 in pMSCs. The expression of the remaining markers were very low to negative including CD105 in pMSCs. The statistical comparison of the levels of these positive markers indicated that except CD73 (*p* = 0.4353), CD146 (*p* = 0.1899), and CD166 (*p* = 0.3762), the expression of CD29 (*p* = 0.0167), CD44 (*p* = 0.0422), CD90 (*p* = 0.0096), and CD105 (*p* = 0.0058) was significantly higher in the UC-MSCs than those in the pMSCs (Fig. 2b).

**Fig. 3:**
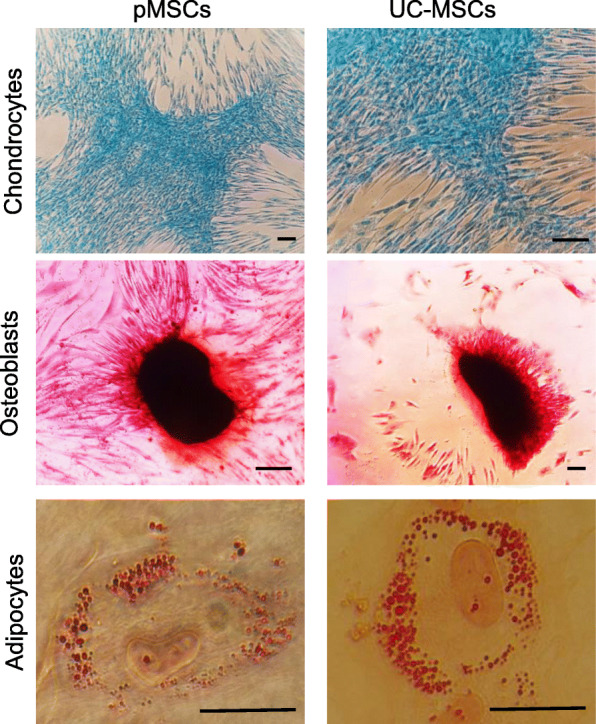
Trilineage differentiation to the chondrocytes, osteocytes, and adipocytes. Data are a typical microscopic view of differentiated cells from one representative experiment, which were repeated three times separately. Bar: 20 μm

The in vitro multipotential differentiation between pMSCs and UC-MSCs was compared and confirmed. As shown in Fig. 3, both pMSCs and UC-MSCs could be induced to osteocytes, adipocytes, and chondrocytes under the same in vitro conditions without any marked difference.

### Different potencies between pMSCs and UC-MSCs in the prevention of hypertonic PDS-induced PM dysfunction and tissue injury

**Fig. 4:**
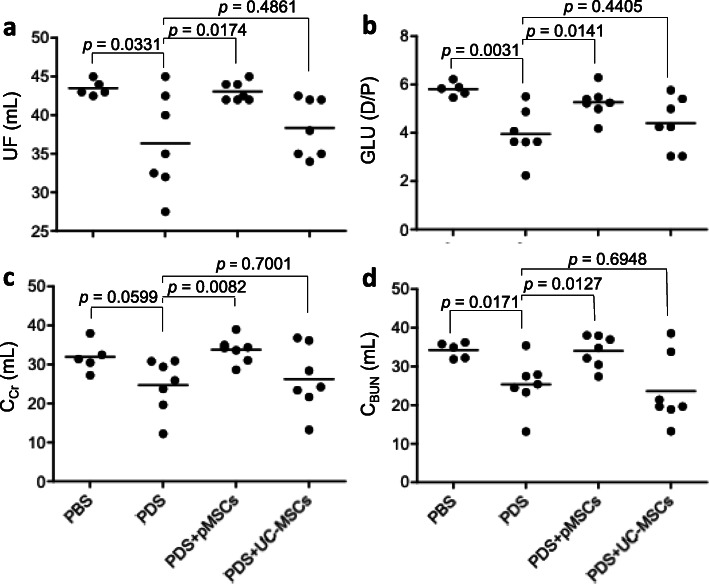
PM function after treatment with either pMSCs or UC-MSCs. The PM function at the experimental endpoint was determined by the measurement of ultrafiltration (UF) (**a**), the glucose dialysate-to-plasma ratio (GLU D/P) (**b**), creatinine clearance (C_Cr_) (**c**), and BUN clearance (C_BUN_) (**d**). The UF of each rat was determined once, and the level of each chemical (GLU, Cr, and BUN) in each sample was measured in triplicate and was represented by the average of these measurements. Data are presented as the mean ± SD of each group (PBS: *n* = 5 rats; PDS: *n* = 7 rats; PDS + pMSCs: *n* = 7 rats; and PDS + UC-MSCs: *n* = 7 rats). The difference between groups was analyzed using two-tailed *t*-test. pMSCs *vs.* UC-MSCs: *p* = 0.0086 (UF), *p* = 0.0908 (GLU D/P), *p* = 0.0482 (C_Cr_), and *p* = 0.0173 (C_BUN_)

The PM injury of 5/6Nx rat was induced by daily exposed to a Dianeal PDS, and some of them received the treatment of either pMSCs or UC-MSCs (2 × 10^6^ cells). As shown in Fig. 4, as compared with PBS control (*n* = 5), the daily IP injection of PDS alone (PDS group, *n* = 7) significantly damaged the function of PM, evidenced by the significantly lower values of UF (*p* = 0.0331), GLU D/P ratios (*p* = 0.0031), C_Cr_ (*p* = 0.0599), and C_BUN_ (*p* = 0.0171). As compared to PDS group, an increase in PM functional parameters was seen in PDS + pMSCs group (*n* = 7)(UF: *p* = 0.0174; GLU D/P: *p* = 0.0141; C_Cr_: *p* = 0.0082; and C_BUN_: *p* = 0.0127), but not in PDS + UC-MSCs group (*n* = 7) that was indicated by no significant differences of the PM parameters between PDS and PDS-UC-MSC groups. The outcomes of pMSC and UC-MSC treatments were different in the UF (*p* = 0.0086), and the clearance of Cr (*p* = 0.0482) and BUN (*p* = 0.0173), but not in the GLU absorption (D/P) (*p* = 0.0908). These data suggested that pMSCs were more potent than UC-MSCs in the prevention of the PDS-induced PM dysfunction

**Fig. 5:**
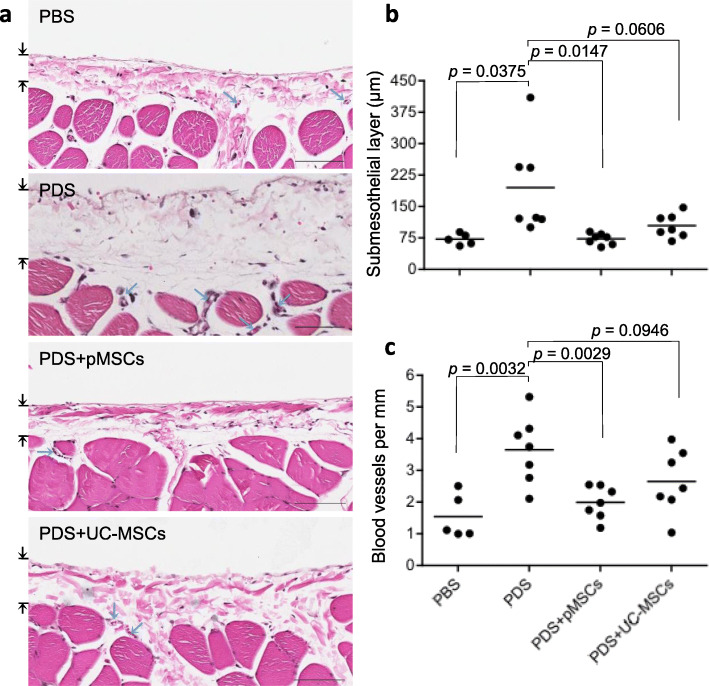
Histologic analysis of the structural damage of the PM. **a** Typical microscopic views of H&E-stained PM for each group. The thickness of submesothelial layer was indicated by the area between two arrows. Blue arrows: blood vessels and capillaries, bar: 100 μm. **b** The thickness of the submesothelial layer in each group (PBS: *n* = 5 rats; PDS: *n* = 7 rats; PDS + pMSCs: *n* = 7 rats; and PDS + UC-MSCs: *n* = 7 rats). Data are presented as the mean ± SD. The difference between groups was analyzed using two-tailed *t*-test. **c** The number of the blood vessels and capillaries within the submesothelial layer (as a measure of neoangiogenesis) in each group (PBS: *n* = 5 rats; PDS: *n* = 7 rats; PDS + pMSCs: *n* = 7 rats; and PDS + UC-MSCs: *n* = 7 rats). Data are presented as the mean ± SD. The difference between groups was analyzed using two-tailed *t*-test. pMSCs *vs.* UC-MSCs: *p* = 0.0208 (submesothelial layer thickness), and *p* = 0.1515 (blood vessel number)

The levels of PM tissue damage, including the thickness of submesothelial layer and the number of blood vessels and capillaries, were examined using histologic analysis. Figure 5a presents a typical microscopic view of the PM tissues in each group, showing the affected submesothelial layer and the blood vessels and capillaries. A semi-quantitative histologic analysis revealed that the submesothelial layer in PDS group (195.14 ± 112.54 μm, *n* = 7) was significantly thicker than that (72.18 ± 13.45 μm, *n* = 5) in PBS group (*p* = 0.0375), suggesting that daily exposure to the PDS induced the thickness of the PM (Fig. 5b). After pMSC treatment, the thickness of the submesothelial layer (73.17 ± 13.15 μm, *n* = 7) was similar to that in the PBS group and was significantly reduced as compared to that in the PDS group (*p* = 0.0147). In contrast to the pMSCs, administration of UC-MSCs did reduce the layer thickness to 104.36 ± 28.10 μm (*n* = 7) but not significantly (*p* = 0.0606) (Fig. 5b) in this limited number of rats. Further analysis showed that the outcome of pMSC treatments was significantly different from that of UC-MSCs (*p* = 0.0208). Similarly to the submesothelial layer, the numbers of the blood vessels and capillaries in PDS group were significantly higher than those in PBS group (*p* = 0.0032), and were decreased by the treatment with pMSCs (PDS vs. PDS + pMSCs, *p* = 0.0029) but not with UC-MSCs (PDS vs. PDS + UC-MSCs, *p* = 0.0946) (Fig. 5c). However, the difference between pMSCs and UC-MSCs in the reduction of neoangiogenesis was not significant (*p* = 0.1515). All these data suggested pMSCs were more effective than UC-MSCs on the protection of the PM from PDS-induced structural modification, but not neoangiogenesis.

### Different potencies between pMSCs and UC-MSCs in the prevention of hypertonic PDS-worsened kidney failure

**Fig. 6:**
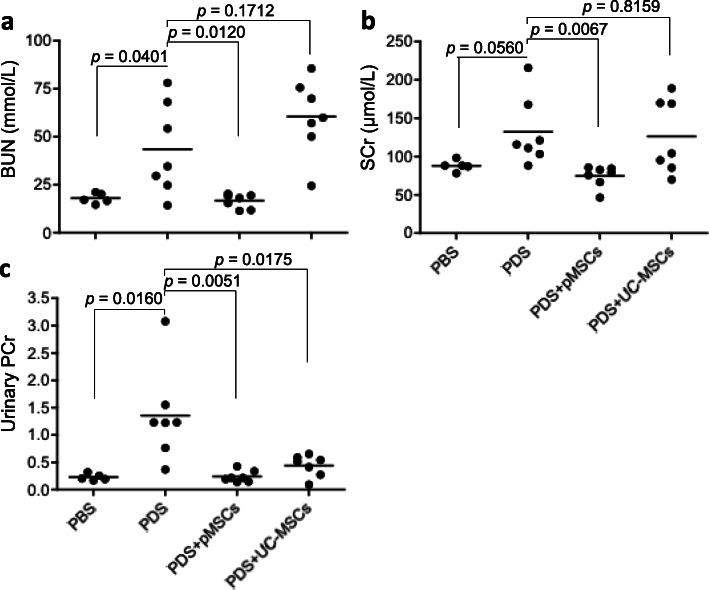
Remnant kidney function after treatment with either pMSCs or UC-MSCs. The remnant kidney function at the experimental endpoint was determined by the measurement of serum BUN (**a**), serum creatinine (SCr) (**b**), and the protein-to-creatinine ratio in urine (urinary PCr). The level of each chemical (BUN, Cr, and protein) in each sample was measured in triplicate and was represented by the average of the measurements. Data are presented as the mean ± SD of each group (PBS: *n* = 5 rats; PDS: *n* = 7 rats; PDS + pMSCs: *n* = 7 rats; and PDS + UC-MSCs: *n* = 7 rats). The difference between groups was analyzed using two-tailed *t*-test. pMSCs *vs.* UC-MSCs: *p* < 0.0001 (BUN), *p* = 0.0184 (SCr), and *p* = 0.0354 (urinary PCr)

Subtotal 5/6Nx in rats is a common experimental model of chronic kidney disease [39]. Thus, the secondary outcome measure of MSC treatments (pMSCs vs UC-MSCs) was their effects on the progression of kidney failure in these rats. As shown in Fig. 6, daily intraperitoneal exposure to the PDS worsened the kidney failure, indicated by the elevated levels of BUN (*p* = 0.0401), SCr (*p* = 0.0560), and PCR in urine (*p* = 0.0160) as compared to those in PBS group. The treatment with pMSCs but not with UC-MSCs significantly prevented the increase in both BUN (*p* = 0.0120) and SCr (*p* = 0.0067) as compared to those in the PDS group (Fig. 6a, b). Interestingly, both types of MSCs suppressed the urinary PCr induced by the exposure to the PDS although the decreased levels were slightly different—from 1.35 ± 0.85 in PDS group to 0.24 ± 0.11 by pMSCs (*p* = 0.0051) or to 0.44 ± 0.20 by UC-MSCs (*p* = 0.0175) (Fig. 6c). Further, the different outcomes between pMSCs and UC-MSC treatments were significant in the reduction of BUN (*p* < 0.0001) and SCr (*p* = 0.0184) and urinary PCr (*p* = 0.0354).

**Fig. 7:**
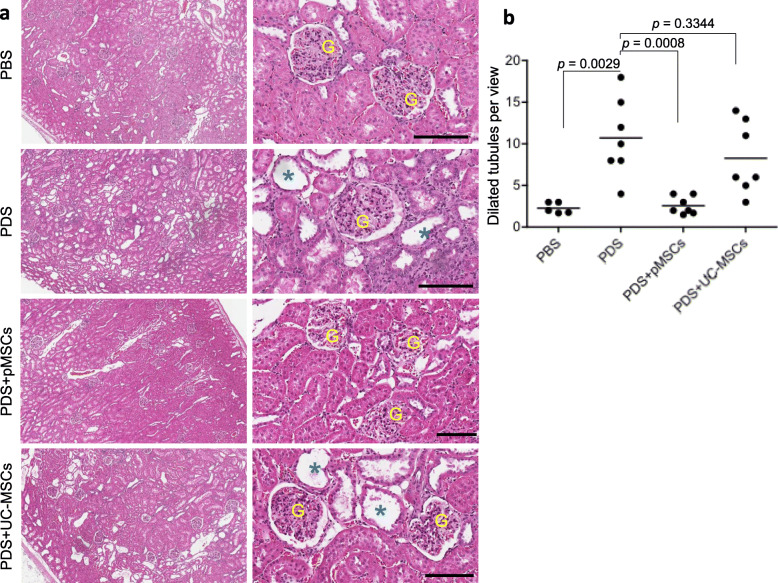
Histologic assessments of kidney tissue damage. **a** Typical microscopic views of the renal cortex (H&E stain) for each group. The left hand column: low power images; the right hand column: high power images. G: glomerulus, *: dilated tubules, Bar: 100 μm. **b** Tissue sections were viewed under × 100 magnification, and the numbers of dilated tubules in the renal cortex were counted. Data are presented as the mean ± SD of each group (PBS: *n* = 5 rats; PDS: *n* = 7 rats; PDS + pMSCs: *n* = 7 rats; and PDS + UC-MSCs: *n* = 7 rats). The difference between groups was compared using two-tailed *t*-test. *p* = 0.0054 (pMSCs *vs.* UC-MSCs)

The additional evidence of MSC treatment for the protection of damaged kidneys was further confirmed by the histologic analysis. As shown in Fig. 7a, tubule dilation was the most noticeable pathological change in the tissue sections. A semi-quantitative histologic assessment of these dilated tubules revealed that the number of the dilated tubules per view in PDS group (10.71 ± 4.72, *n* = 7) was significantly more than that (2.30 ± 0.65, *n* = 5) in PBS group (*p* = 0.0029). Furthermore, the treatment with pMSCs significantly prevented the tubule dilation in the kidneys as the less dilation (2.60 ± 1.07) in this group than that in the PDS (*p* = 0.0008, *n* = 7), which was not seen in the kidneys of the rats receiving UC-MSCs (*p* = 0.3344) (Fig. 7b). Again, the difference of tubular damage between pMSCs and UC-MSCs group was significant (*p* = 0.0054).

### Difference in susceptibility to uremic toxins between pMSCs and UC-MSCs

**Fig. 8:**
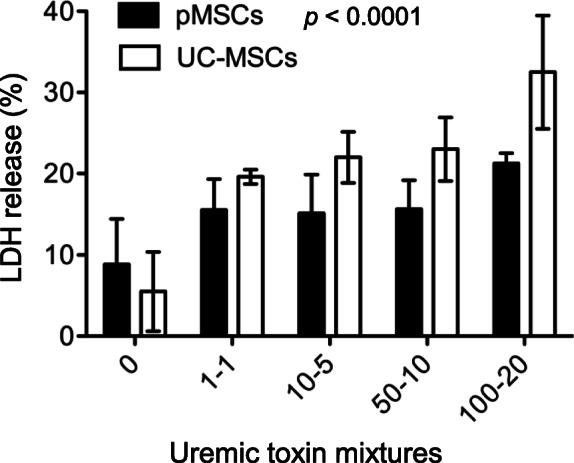
Different sensitivity of between pMSCs and UC-MSCs to the cytotoxicity of a panel of uremic toxins. A monolayer of pMSCs or MC-MSCs was incubated with different concentrations of a panel of common uremic toxins (Suppl Table 3) for 24 h. The cell death of MSCs was determined by LDH release. The LDH level in each sample per an experiment was measured in triplicate and was represented by the average of the measurements. Data are presented as the mean ± SD of each group (0, *n* = 4 samples; 1-1: *n* = 6 samples; remaining groups: *n* = 8 samples). The difference between pMSCs and UC-MSCs was compared using two-way ANOVA

When the kidneys fail, a variety of uremic toxins are accumulated in the patient’s body [40], suggesting that MSCs that have potential for use as a therapy for ESRD patients need to survive under the uremic toxin environment. LDH release as a biomarker of cell death was measured in cultured pMSCs and UC-MSCs following incubation with different concentrations of a panel of uremic toxin (Suppl. Table 3). As shown in Fig. 8, there was more cell death in UC-MSCs than pMSCs after incubation with the same amounts of uremic toxins (*p* < 0.0001, two-way ANOVA). For example, the incubation with 100-20 mixture of uremic toxins induced 32.5 ± 6.97% of LDH release from UC-MSCs as compared to 21.25 ± 1.28% from pMSCs (*p* = 0.0005, *n* = 8). These data suggested that pMSCs were more resistant than UC-MSCs to the toxicity of these uremic toxins found in the body of PD patients.

### Different cytoprotection and NOS 2 inhibition of secretome from pMSCs and UC-MSCs in vitro

**Fig. 9:**
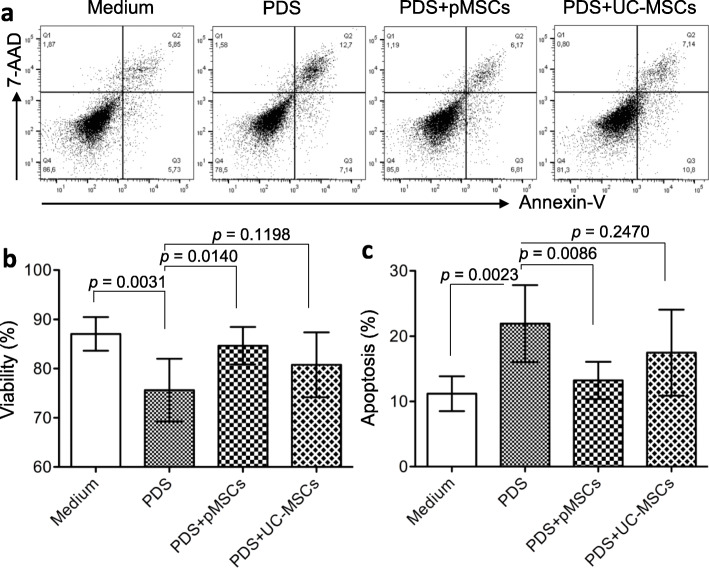
Different protection of HPMCs from PDS-induced cell death by between pMSCs-CM and UC-MSCs-CM. A monolayer of HPMCs was exposed to 4.25% dextrose Dianeal PDS for 20 min, followed by 24-h incubation with either pMSCs-CM or UC-MSCs-CM. Cell viability/survival or apoptosis was measured using FACS analysis with staining with both 7-AAD and Annexin-V. **a** A typical FACS graph showing cell viability and cell death in each group. Q1: 7-AAD positive—necrotic cells; Q2 and Q3: Annexin-V positive—apoptotic cells; and Q4: both negative—viable cells. **b** Viability or survived cells in Q4. **c** Apoptosis (total of Q2 and Q3). The cell viability or apoptosis in each sample per an experiment was determined in duplicate and was represented by the average of the measurements. Data are presented as the mean ± SD of each group (*n* = 6 samples). The difference between groups was analyzed using two-tailed *t*-test

There are various pathways responsible for therapeutic potential of MSCs including their secretome [41, 42]. In this study, the effects of the secretome, derived from both pMSCs and UC-MSCs, on the survival of HPMCs following exposure to a PDS and NOS 2 activity, an inflammatory mediator, of activated monocytes (THP-1 cells) were compared. A typical graph from each group showed the survived/viable (Q4: double negative population) and apoptotic cells (total of Q2 and Q3: Annexin-V staining positive) in FACS analysis (Fig. 9a). A 20-min exposure to the 4.35% dextrose Dianeal PDS significantly reduced the viability of cultured HPMCs from 87.07 ± 3.4% in medium to 75.63 ± 6.39% in PDS-treated group (*p* = 0.0031, *n* = 6) (Fig. 9b) or induced the apoptosis from 11.19 ± 2.68% to 21.93 ± 5.89% (*p* = 0.0023, *n* = 6) (Fig. 9c). The PDS-induced cell death was significantly prevented by the supernatant from pMSCs, in which the cell viability in the PDS was increased to 84.65 ± 3.83% in PDS + pMSCs (*p* = 0.0140, *n* = 6) (Fig. 9b), or the cell apoptosis from the PDS group was decreased to 13.23 ± 2.87% (*p* = 0.0086, *n* = 6) (Fig. 9c). However, the cytoprotection of the supernatant from UC-MSCs was not as significant as that from pMSCs in the prevention of PDS-induced cell death of cultured HPMCs.

**Fig. 10:**
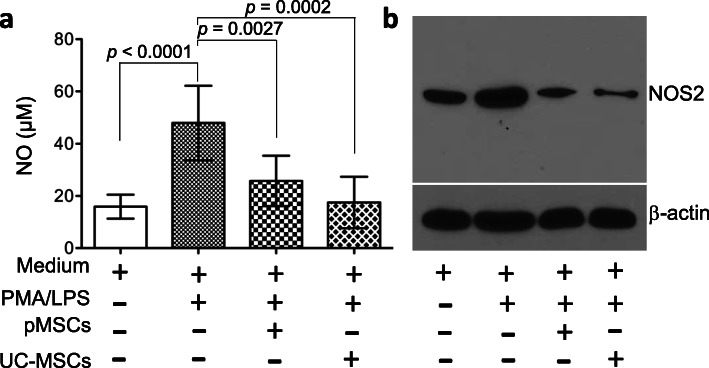
Different suppression of NOS 2 expression and activity in activated THP-1 by between pMSCs-CM and UC-MSCs-CM. THP-1 monocytes in cultures were activated by the addition of PMA for 24 h, followed by the additional 24 h with LPS. The activated THP-1 cells were incubated with either pMSCs-CM or MU-MSCs-CM. **a** Nitric oxide (NO) levels from Griess assay. The NO level in each sample per an experiment was measured in triplicate and was represented by the average of the measurements. Data are presented as the mean ± SD of each group (*n* = 8 samples). The difference between groups was analyzed using two-tailed *t*-test. **b** NOS 2 protein levels in Western blot analysis. Data are representative of three independent experiments

The therapeutic actions of the secretome derived from pMSCs and UC-MSCs were further compared in the inhibition of NOS 2 expression and activity in the activated monocytes. As shown in Fig. 10, following activation of THP-1 by incubation with PMA and LPS, THP-1 cells upregulated NOS 2 protein level and produced an abundant amount of NO in the medium, from 15.88 ± 4.61 μM in the medium alone to 47.88 ± 14.28 μM in the medium containing PMA/LPD (*p* < 0.0001, *n* = 8). The upregulation of NOS 2 protein and NO production by the stimulation with PMA/LPS were significantly inhibited by the supernatants from both pMSC (NO levels: 25.75 ± 9.65 μM, PMA/LPS vs PMA/LPS + pMSCs, *p* = 0.0027, *n* = 8) and UC-MSC (NO levels: 17.5 ± 9.87 μM, PMA/LPS vs PMA/LPS + UC-MSCs, *p* = 0.0002, *n* = 8) cultures (Fig. 10). These data implied that the secretome derived from pMSCs and UC-MSCs might have similar capacity in the suppression of NOS 2 activity in the inflammatory monocytes/macrophages.

## Discussion

There has been a very broad interest in clinical application of MSCs and their products (e.g., extracellular vesicles or secretome) for tissue damage repair, fibrosis prevention, immunomodulation, and anti-inflammation in various pathologies, including the peritoneal inflammation and fibrosis (see recent reviews: [29, 43–45]). At present, the MSCs in clinical studies are mainly isolated from the BM, adipose, or perinatal (e.g., UC and placenta) tissues [46]. However, the selection of using autologous MSCs from a patient’s own body has several advantages over allogeneic MSCs such as availability, no need of HLA matching and/or immunosuppression, and no risk of graft-versus-host disease, but there are practical limitations to extraction of these cells such as the difficulty and invasiveness of the procurement [47]. We for the first time isolated the MSCs from otherwise “discarded” PD fluids—pMSCs [24]. The clinical use of these autologous pMSCs specifically for controlling peritoneal inflammation or preserving PM structure during PD in PD patients has all the benefits as mentioned above, plus the unlimited availability without any additional “invasive” harvest. We previously demonstrated the effectiveness of pMSCs on the protection of PM from Dianeal PDS (4.25% glucose)-induced morphological alteration, neoangiogenesis and functional loss (fluid removal) in healthy rats [30]. To further confirm the feasibility of using pMSCs for prevention of PDS-induced peritoneal inflammation in PD patients, in this study we compared the effectiveness of the pMSCs with that of UC-MSCs on the PDS-induced peritoneal inflammation in uremic rats. The in vivo results revealed that pMSCs were more effective than UC-MSCs in the protection of both the PM and remnant kidney from PDS (4.25% dextrose Dianeal)-initiated damage in the preclinical rat model. These data were supported by the more survived pMSCs than UC-MSCs in the presence of uremic toxins and the higher activity of the secreted bioactive factors (secretome) from pMSCs than those from UC-MSCs in the prevention of PDS-induced peritoneal mesothelial cell death. Interestedly, the expression levels of stemness and MSC key markers of perinatal UC-MSCs were higher than those in adult tissue-sourced pMSCs, which were correlated with a higher growth rate of UC-MSCs than that of pMSCs (data not shown) but not with their cytoprotective activities in both in vitro and in vivo.

In general, the survival and potency are two essential requirements for the in vivo effectiveness of administered human MSCs in rats. The survival or fate of grafted human MSCs in host rodents may depend on the site or microenvironment of cell injection, where the immune response to “foreign” antigens is different. It has been reported that some human UC-MSCs after injection to the brain or BM cavity of rats could survive for 1 to 2 months [48–50]. However, the presence of human BM-MSCs is not detectable after 1 week of injection into the myocardium where there is a significant macrophage infiltration in the rats [51]. After human BM-MSCs injected into the peritoneal cavity of immunocompetent mice, these human cells initially aggregate with macrophages but disappear within a week [52]. Similarly, IP injected rat BM-MSCs into isogeneic rat recipients cannot be found in the peritoneal mesothelium after 3 days of transplantation [53]. In both present and previous studies [30], we could not localize any of injected human cells (both pMSCs and UC-MSCs) in the PM sections by immunohistochemical staining of human nuclear antigen at the experimental endpoint (at day 7 after the last injection) (data not shown). All these studies may suggest that the MSCs regardless of their source only survive for a short period of time (no longer than a week) in the peritoneal cavity of rats. We did not compare the immunophenotypic profile of pMSCs with UC-MSCs in the present study, but UC-MSCs are more sensitive than pMSCs in cultures to the cytotoxicity of uremic toxins that are slowly accumulated in the body with kidney failure (Fig. 8). This observation may imply that the survival of pMSCs may be longer such as a few more hours than that of UC-MSCs in uremic rats after IP injection, which however needs further investigation.

Therapeutic actions of MSCs during a MSC-based therapy are complex and may be differently affected at the different site or environment of the body [54–56], which mainly include tissue protection by cytoprotection and differentiation into multiple cell lineages and regulation of immune responses via immunomodulation. The potential for cell trilineage differentiation in vitro is similar between pMSCs and UC-MSCs (Fig. 2), and no evidence is seen that these human cells differentiate into peritoneal mesothelial cells in rats. Although the mechanism of MSCs’ action has not been completely understood, numerous studies have demonstrated the extracellular secreted factors and vesicles—their secretome as their “active ingredients” for regenerative and cytoprotective effects, and immunomodulatory property of the MSC-based therapy [57, 58]. MSC-derived soluble factors and vehicle-bound substances (secretome or conditional medium) from different sources consist of IFN-γ, IL-17, IL-10, IL-6, TGF-β, IL-2, TNF-α, and HLA (for immunomodulation), and GDNF, FGF, IGF, BDNF, PEGF, PDGF, miRNAs, VEGF, and MMPs (for cell survival/growth and tissue remodeling) [59–61]. The composition of the secretome of MSCs appears to vary significantly, depending on the age of the host and niches where the cells are collected [60, 61]. In this study, we did not investigate the differences of the secreted factors (secretome) between adult pMSCs and UC-MSCs from newborn babies, but other studies show that as compared with UC-MSCs, the adult AD-MSCs produce more growth factors (e.g., VEGF, insulin-like growth factor, bFBF) and SDF-1 or less pro-apoptotic thrombospondin-1 [62–64]. As shown in Fig. 9, the secretome of pMSCS is more protective than that of UC-MSCs against a PDS-induced peritoneal mesothelial cell death, which suggested that the secretome from adult pMSCs may have more or less the same levels of cytoprotective molecules as seen in adult AD-MSCs.

There are a large number of infiltrating macrophages in the parietal peritoneum biopsies from the pre-dialysis stage, which is specifically associated with higher PD technique failure and mortality in PD patients [65, 66], suggesting that macrophages play an important role in the initiation of peritoneal inflammation in PD patients. After IP injection, human MSCs immediately aggregate with macrophages [52], and the macrophages are the target of MSCs for their immunoregulatory property via both active and passive mechanisms—both complicated [56]. The active mechanisms are via secretion of soluble factors such as TSG-6, PGE2, and lactate and others (e.g., TGF-β3, thrombospondin-1, IL-6) that drives M1 proinflammatory phenotype of macrophages toward an M2 anti-inflammatory phenotype for inflammation suppression or tissue repair [56, 67–69]. The passive mechanisms of MSCs in regulation of macrophages include the intracellular signaling regulators, such as miRNA in secretome and mitochondria which are shuttled to the cell membrane of MSCs during mitophage [56]. The miRNAs downregulate Toll-like receptor signaling and upregulate cytokine production (e.g., IL-10), while phagocytosis of mitochondria from MSCs improved the bioenergetics of macrophages [56]. Using a PCR array analysis, we have demonstrated that pMSC-derived CM or secretome downregulate the proinflammatory gene expression (i.e., CXCL6, NOS 2, IL1RN, CCL5, and NR3C1), while they upregulate anti-inflammatory genes (i.e., CCR1, CCR4, IL-9, and IL-10) in activated macrophages [30]. Further, the inhibition of NOS 2 expression or activity by both pMSC- and UC-MSC-derived CM was similar (Fig. 10). However, whether there is any difference between adult pMSCs and UC-MSCs in both active and passive regulatory pathways of macrophages needs further investigation. It has been shown that AD-MSCs secret less proinflammatory mediators such as MIP-2 (13.05 fold), IL-6 (6.84 fold), GRO (4.73 fold), MMP-1 (3.19 fold), and IL-8 (3.08 fold) than UC-MSCs [64]. Among them, MIP-2, GRO and IL-8 induces neutrophil rolling and extravascular migration [70, 71], suggesting that adult AD-MSCs are more effective and efficient than UC-MSCs for the control of inflammatory response. Indeed, in a rat model of spinal cord injury AD-MSCs transplants decrease TNF-a or upregulate IL-10 more significantly than transplanted UC-MSCs in spinal tissues [72]. In this study, adult pMSCs were also more effective than UC-MSCs in the reduction of inflammation in both local PM and remnant kidneys in the rat model of PD, suggesting that both adult AD-MSCs and pMSCs may attenuate the inflammatory response in a similar manner, which however needs further investigation.

The limitation of this study was largely related to the experimental model. First, the pathogenesis of PM injury in uremic rat model may not be the same as in PD patients although both are uremic and exposed to a hypertonic PDS. Second, the possible occurrence of xeno-immunity stimulated by human MSCs in rats could affect their therapeutic actions such as survival. Third, human MSCs may exhibit different biological functions in different hosts—rats versus humans, particularly in the situation of using pMSCs as cell autotransplantation and UC-MSCs as cell allotransplantation. In addition, the molecule mechanisms underlying the cytoprotection and immunoregulatory functions of both pMSCs and UC-MSCs are not investigated. For example, which secreted molecule(s) from MSCs protect mesothelial cells from apoptosis, and which inactivate macrophages or switch macrophage phenotype from M1 (an inflammatory mediator) to M2 (a tissue healing mediator), and the mechanisms by which the pMSCs-CM are more protective than UC-MSCs-CM in the situation of PDS-induced mesothelial cell death.

## Conclusions

Recently, there has been an increasing interest in using MSCs to protect the PM of PD patients from injury during PD. IP injection of rat BM-MSCs or human AD-MSCs and pMSCs significantly reduces PDS-induced submesothelial thickness and cellular infiltration of the PM [30, 73, 74]. The present study demonstrates the different effectiveness between pMSCs and UC-MSCs in the prevention of PDS-induced peritoneal alterations including peritoneal thickening and neoangiogenesis that associate with protection of UF (both fluid removal, and BUN and Cr clearance), and in the preservation of remnant kidney function. Taken together, all these findings may provide preclinical evidence for a novel approach by using MSCs, particularly pMSCs from PD patient’s own body, in the treatment of PM alternations and residual kidney failure in PD patients.

## Supplementary Information

**Additional file 1.**

**Additional file 2.**

**Additional file 3.**

## Data Availability

All the data used to support the findings of this study are included within the article and are also available from the corresponding author (Caigan Du) upon request.

## Abbreviations

7-AAD: 7-Amino-actinomycin D
AD: Adipose tissue
ANOVA: Analysis of variance
BM: Bone marrow
BUN: Blood urea nitrogen
CM: Conditional medium
Cr: Creatinine
Ct: Threshold cycle
DMEM/F1: Dulbecco’s modified Eagle’s medium/Ham’s nutrient mixture F12
DMSO: Dimethylsulfoxide
D/P: Dialysate-to-plasma ratio
ESRD: End-stage renal disease
FACS: Fluorescence-activated cell sorting
FBS: Fetal bovine serum
GLU: Glucose
H&E: Hematoxylin and eosin
HPMCs: Human peritoneal mesothelial cells
IP: Intraperitoneal injection
LDH: Lactate dehydrogenase
LPS: Lipopolysaccharides
MFI: Mean fluorescence intensity
MSCs: Mesenchymal stromal cells
NO: Nitric oxide
NOS 2: Nitric oxide synthase 2
Nx: Nephrectomized
P4: Four passages
PAGE: Polyacrylamide gel electrophoresis
PBS: Phosphate buffered saline
PCr: Protein-to-creatinine ratio
PD: Peritoneal dialysis
PDS: Peritoneal dialysis solution
PE: Phycoerythrin
PM: Peritoneal membrane
PMA: Phorbol 12-myristate 13-acetate
pMSCs: Peritoneal dialysis effluent-derived mesenchymal stromal cells
RPMI: Roswell Park Memorial Institute
RRF: Residual renal function
RT-qPCR: Quantitative reverse transcription-polymerase chain reaction
SCr: Serum creatinine
SD: Standard deviation
UC: Umbilical cord
UFF: Ultrafiltration failure
