# Supplementary material for: Comparison of mesenchymal stromal cells from peritoneal dialysis effluent with those from umbilical cords: characteristics and therapeutic effects on chronic peritoneal dialysis in uremic rats
Source: Stem Cell Res Ther. 2021 Jul 13;12:398. doi: 10.1186/s13287-021-02473-9 (PMC8278755; doi:10.1186/s13287-021-02473-9)
Supplement: Supplementary file 1 — Additional file 1. [file 13287_2021_2473_MOESM1_ESM.docx]

**Suppl. Table 1** The RT-qPCR primer sequences of stemness marker genes

| Gene | Forward (5’ to 3’) | Reverse (5’ to 3’) |
| --- | --- | --- |
| *NANOG* | TGAACCTCAGCTACAAACAG | TGGTGGTAGGAAGAGTAAAG |
| *C-MYC* | ACTCTGAGGAGGAACAAGAA | TGGAGACGTGGCACCTCTT |
| *KLF4* | TCTCAAGGCACACCTGCGAA | TAGTGCCTGGTCAGTTCATC |
| *OCT4* | GACAGGGGGAGGGGAGGAGCTAGG | CTTCCCTCCAACCAGTTGCCCCAAA |
| *SOX2* | AGCTACAGCATGATGCAGGA | GGTCATGGAGTTGTACTGCA |
| *LIN28* | TGCGGGCATCTGTAAGTGG | GGAACCCTTCCATGTGCAG |
| *CCNA2* | CTGGTGGTCTGTGTTCTGTGA | TGCCAGTCTTACTCATAGCTGA |
| *MCM6* | CCAGTGGTAAAGCGTCCAGT | CCACTGATTGGGTTTGCTGC |
| *RAD21* | GCTGCCGCAAAGTTCTACAG | GGTCCAGGTGTTGCGATGAT |
| *EXO1* | AAACCTGAATGTGGCCGTGT | CCTCATTCCCAAACAGGGACT |
| *STAG1* | ACAGATGCTGTACACCGTTCA | GGCAGGGCATGAAATAAGTGG |
| *β-Actin* | CTGGCACCACACCTTCTACAATG | AATGTCACGCACGATTTCCCGC |

The sequences of the primers were from literature [1, 2], and were synthesized in Invitrogen (Thermo Fisher Scientific).

Reference:

1. Kim D, Kim C-H, Moon J-I, et al., Generation of human induced pluripotent Stem cells by direct delivery of reprogramming proteins. Cell Stem Cell, 2009, 4(6): 472-6.
2. Ruan J, Shen J, Wang Z, et al., Efficient preparation and labeling of human induced pluripotent stem cells by nanotechnology. Int J Nanomedicine. 2011, 6: 425-35.
