# Supplementary material for: Comparison of mesenchymal stromal cells from peritoneal dialysis effluent with those from umbilical cords: characteristics and therapeutic effects on chronic peritoneal dialysis in uremic rats
Source: Stem Cell Res Ther. 2021 Jul 13;12:398. doi: 10.1186/s13287-021-02473-9 (PMC8278755; doi:10.1186/s13287-021-02473-9)
Supplement: Supplementary file 2 — Additional file 2. [file 13287_2021_2473_MOESM2_ESM.docx]

**Suppl. Table 2** Monoclonal antibodies for detection of MSCs surface markers

| Marker name | Monoclonal antibody and clone name | Vendor |
| --- | --- | --- |
| CD14 | APC-mouse anti-human CD14 monoclonal antibody, clone 61D3 | *e*Bioscience |
| CD29 | APC-mouse anti-human CD29 monoclonal antibody, clone TS2/16 | BioLegend |
| CD34 | APC-mouse anti-human CD34 monoclonal antibody, clone 4H11 | *e*Bioscience |
| CD44 | APC-rat anti-human/mouse CD44 monoclonal antibody, clone IM7 | *e*Bioscience |
| CD45 | APC-mouse anti-human CD45 monoclonal antibody, clone H130 | BD Biosciences |
| CD73 | PE-mouse anti-human CD73 monoclonal antibody, clone AD2 | BD Biosciences |
| CD79a | PE-mouse anti-human CD79a monoclonal antibody, clone HM47 | *e*Bioscience |
| CD90 | FITC-mouse anti-human CD90 (Thy-1) monoclonal antibody, clone eBio5E10 | *e*Bioscience |
| CD105 | FITC-mouse anti-human CD105 (Endoglin) monoclonal antibody, clone 266 | BD Biosciences |
| CD146 | PE-mouse anti-human CD146 monoclonal antibody, clone P1H12 | BD Biosciences |
| CD166 | PE-mouse anti-human CD166 (ALCAM) monoclonal antibody, clone 3A6 | *e*Bioscience |
| CD271 | PE -mouse anti-human CD271 monoclonal antibody, clone C40-1457 | BD Biosciences |
| HLA-DR | FITC-mouse anti-human HLA-DR monoclonal antibody, clone L243 | *e*Bioscience |
| SSEA-4 | FITC-mouse anti-SSEA-4 monoclonal antibody, clone MC813-70 | BD Biosciences |
| Stro-1 | FITC-mouse anti-human Stro-1 monoclonal antibody, clone MOPC-104E | BioLegend |

APC: allophycocyanin; FITC: fluorescein isothiocyanate; PE: phycoerytrin. Vendor: *e*Bioscience (San Diego, CA, USA), BD Biosciences (Mississauga, ON, Canada), and BioLegend (San Diego, CA, USA)
