# Supplementary material for: Comparison of mesenchymal stromal cells from peritoneal dialysis effluent with those from umbilical cords: characteristics and therapeutic effects on chronic peritoneal dialysis in uremic rats
Source: Stem Cell Res Ther. 2021 Jul 13;12:398. doi: 10.1186/s13287-021-02473-9 (PMC8278755; doi:10.1186/s13287-021-02473-9)
Supplement: Supplementary file 3 — Additional file 3. [file 13287_2021_2473_MOESM3_ESM.docx]

**Suppl Table 3** The composition and concentrations of uremic toxins in mixture solutions

| Mixtures | P-Cresol  (mg/L) | Hippuric acid  (mg/L) | Indoxy sulfate  (mg/L) | Trimethylamine-N-oxide  (mg/L) | Dimethylamine  (mg/L) | Uric acid  (mg/L) |
| --- | --- | --- | --- | --- | --- | --- |
| 100-20 | 100 | 100 | 100 | 20 | 20 | 100 |
| 50-10 | 50 | 50 | 50 | 10 | 10 | 50 |
| 10-5 | 10 | 10 | 10 | 5 | 5 | 10 |
| 1-1 | 1 | 1 | 1 | 1 | 1 | 1 |

The information on uremic toxins was derived from literature (Reference: Vanholder R, De Smet R, Glorieux G, Argiles A, et al. Review on uremic toxins: classification, concentration, and interindividual variability. Kidney Int. 2003;63:1934-43).
